# Involuntary Micturition in Children

**Published:** 1902-03

**Authors:** 


### Involuntary Micturition in Children.

A recent number of *Pediatrics* contains a very excellent paper under the above title, by Dr. G. Frank Lydston, of Chicago.. With reference to the treatment, the author believes that the use of the urethral sound will accomplish much good in a great majority of cases. It corrects vesical hyperesthesia, relieves congestion of the mucous membrane of the deep urethra, and in purely neurotic cases it stimulates the relaxed sphincter to contract. Systematic sounding cures many very obstinate cases. To begin with, of course all sources of reflex irritation should be relieved. Next to the sound the best method of treatment in purely neurotic cases consists in the deep injection of strychnia into the lumbar muscles.

